# Supplementary material for: Targeted Isolation of Rubrolides from the New Zealand Marine Tunicate Synoicum kuranui
Source: Mar Drugs. 2020 Jun 27;18(7):337. doi: 10.3390/md18070337 (PMC7401252; doi:10.3390/md18070337)
Supplement: Supplementary file 1 [file marinedrugs-18-00337-s001.pdf]

marine drugs

MDPI

Supplementary Materials:

Targeted Isolation of Rubrolides from the New Zealand Tunicate *Synoicum kuranui*

Joe Bracegirdle [1,2,3], Luke J. Stevenson [2,3,4], Michael J. Page [5], Jeremy G. Owen [2,3,4], Robert A. Keyzers [1,2,3,*]

[1] School of Chemical and Physical Sciences, Victoria University of Wellington, Wellington 6012, New Zealand; joe.bracegirdle@vuw.ac.nz (J.B.)

[2] Centre for Biodiscovery, Victoria University of Wellington, Wellington 6012, New Zealand

[3] Maurice Wilkins Centre for Molecular Biodiscovery, Wellington 6012, New Zealand

[4] School of Biological Sciences, Victoria University of Wellington, Wellington 6012, New Zealand luke.stevenson@vuw.ac.nz (L.J.S.); jeremy.owen@vuw.ac.nz (J.G.O.)

[5] National Institute of Water & Atmospheric Research (NIWA), P.O. Box 893, Nelson, New Zealand mike.page@niwa.co.nz (M.J.P.)

* Correspondence: robert.keyzers@vuw.ac.nz; Tel.: +64 4 4635117 (R.A.K.)

Figure S1 – Full molecular network of Pacific tunicates 2
Figure S2 – Underwater photos of *Synoicum kuranui* 3
Scheme S1 – Isolation procedure 3
Figure S3 – ^{1}H NMR spectrum screen of the screening fraction 4
Table S1 – NMR data for Rubrolide T (**3**) 5
Figure S4 – ^{1}H NMR Spectrum of **3** 6
Figure S5 – ^{13}C NMR Spectrum of **3** 7
Figure S6 – HSQC NMR Spectrum of **3** 8
Figure S7 – HMBC NMR Spectrum of **3** 8
Figure S8 – (–)-HRESIMS spectra of **3** 9
Figure S9 – (–)-HRESIMS/MS spectrum of **3** 9
Figure S10 – UV/Vis spectrum of **3** 10
Table S2 – NMR data for *Z*–Rubrolide U (*Z*–**4**) 11
Table S3 – NMR data for *E*–Rubrolide U (*E*–**4**) 12
Figure S11 – ^{1}H NMR Spectrum of **4** 13
Figure S12 – ^{13}C NMR Spectrum of **4** 14
Figure S13 – COSY NMR Spectrum of **4** 15
Figure S14 – ROESY NMR Spectrum of **4** 15
Figure S15 – HSQC NMR Spectrum of **4** 16
Figure S16 – HMBC NMR Spectrum of **4** 16
Figure S17 – (–)-HRESIMS spectra of **4** 17
Figure S18 – (–)-HRESIMS/MS spectrum of **4** 17
Figure S19 – UV/Vis spectrum of **4** 18

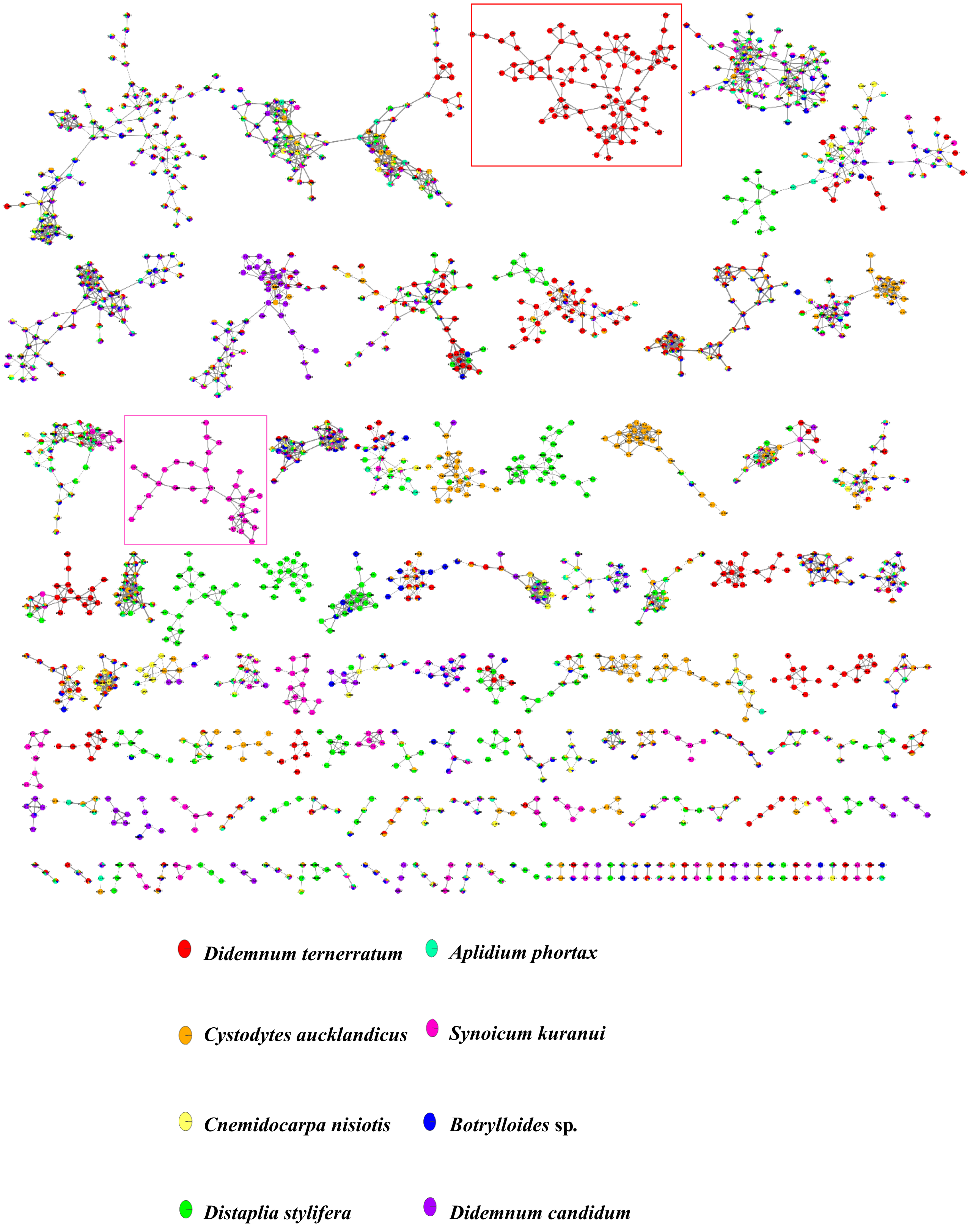

Figure S1 – Full molecular network of Pacific tunicates using both positive and negative ionisation mode HRESIMS. Pink box represents rubrolide-containing constellation A. Red box represents cluster of lamellarin-type molecules.[14] Data can be accessed at https://gnps.ucsd.edu/ProteoSAFe/status.jsp?task=cc4df57385d8478a824e956720a75d04.

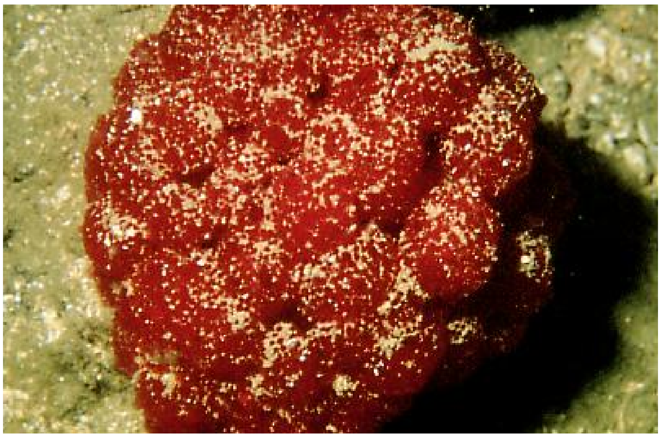

Figure S2 – Underwater photo of the tunicate *Synoicum kuranui* (NIWA ID 101234).

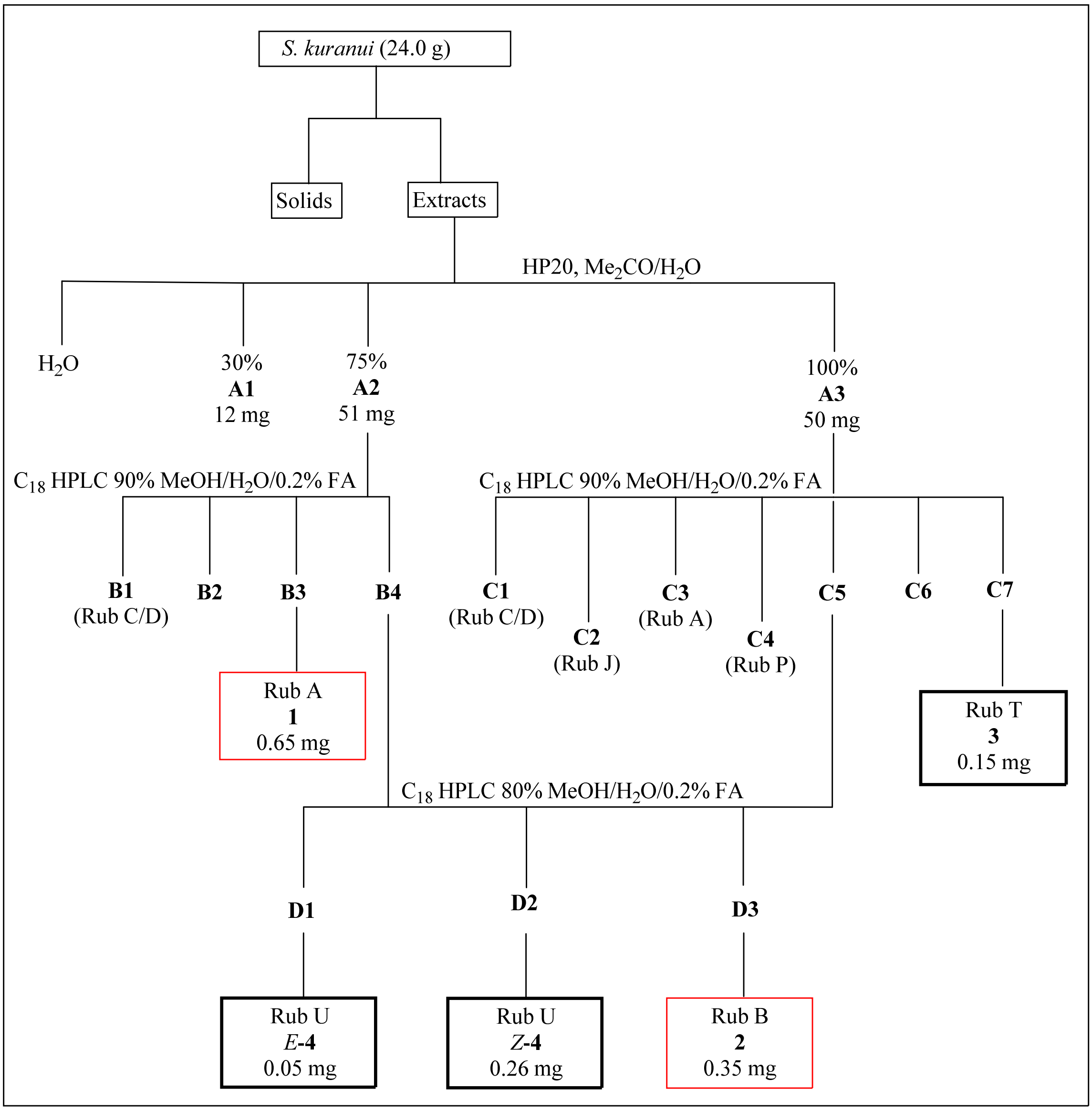

Scheme S1 – Isolation procedure for compounds from *S. kuranui*. Red boxes are previously reported compounds, black bold boxes are new compounds. Under each fraction is a tentative assignment of the previously reported rubrolide present based on the *m*/*z*.

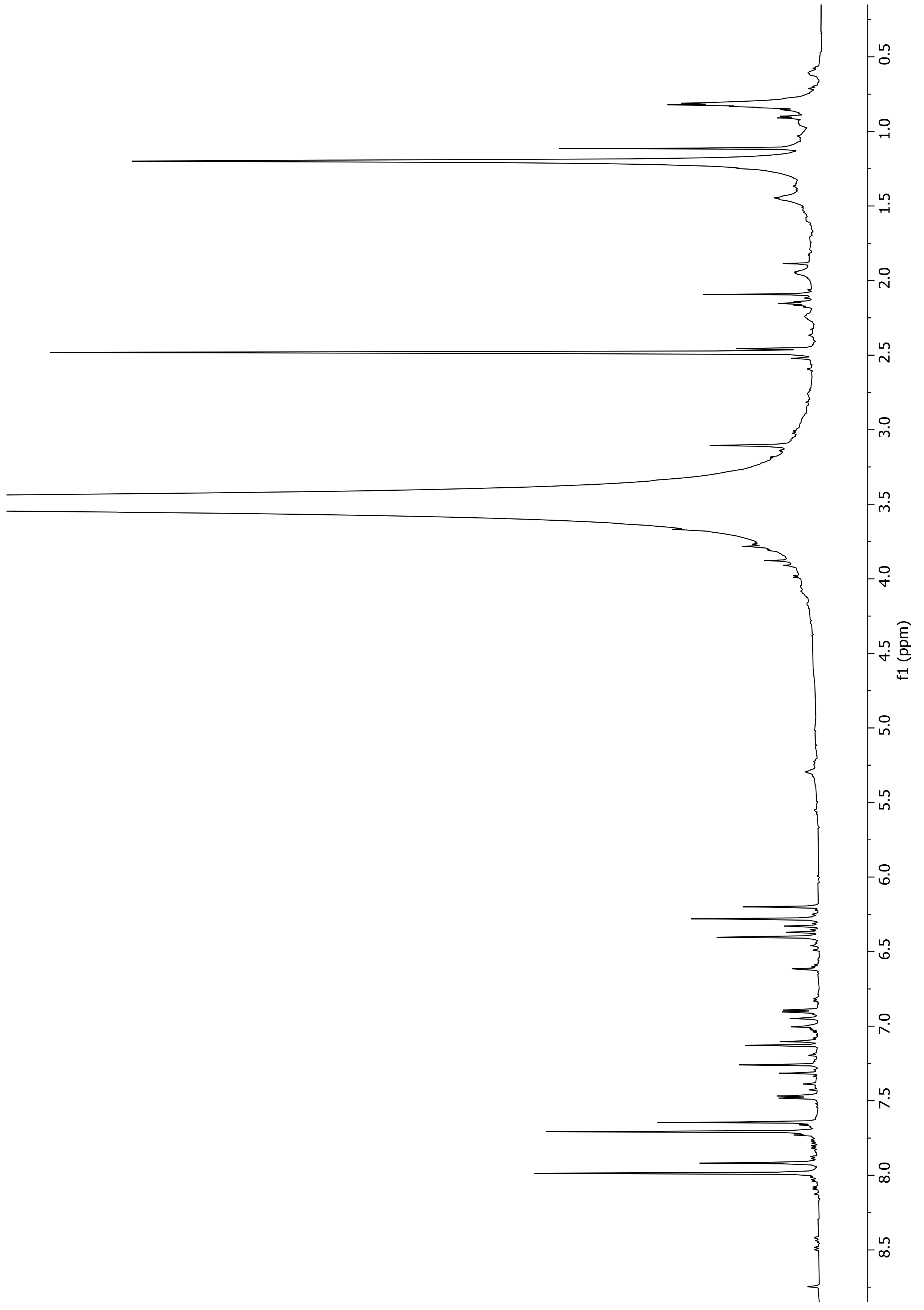

Figure S3 – ^{1}H NMR spectrum screen of *S. kuranui* 75% acetone screening fraction (600 MHz, DMSO–d_6).

Table S1 – ^{13}C (150 MHz) and ^{1}H (600 MHz) NMR data for Rubrolide T (**3**) in DMSO–d_6.

position	^{13}C (δ)	^{1}H (δ, int., mult.)	HMBC
2	168.5		
3	105.7	6.21 (1H, s)	2, 4, 5
4	156.7		
5	148.8		
6	108.2	6.44 (1H, s)	4, 5, 2''/6''
1'	129.9		
2'/6'	131.9	7.59 (2H, s)	4, 2'/6', 3'/5' 4'
3'/5'	115.8		
4'	164.9		
1''	133.0		
2''/6''	134.2	8.15 (2H, s)	6, 2''/6'', 3''/5'', 4''
3''/5''	117.7		
4''	153.1		
CH_3O–4''	60.6	3.83 (3H, s)	4''

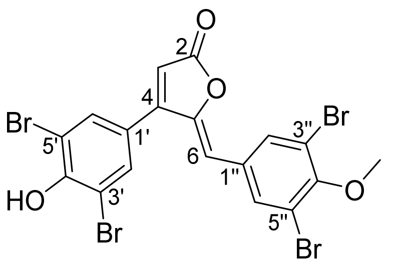

3

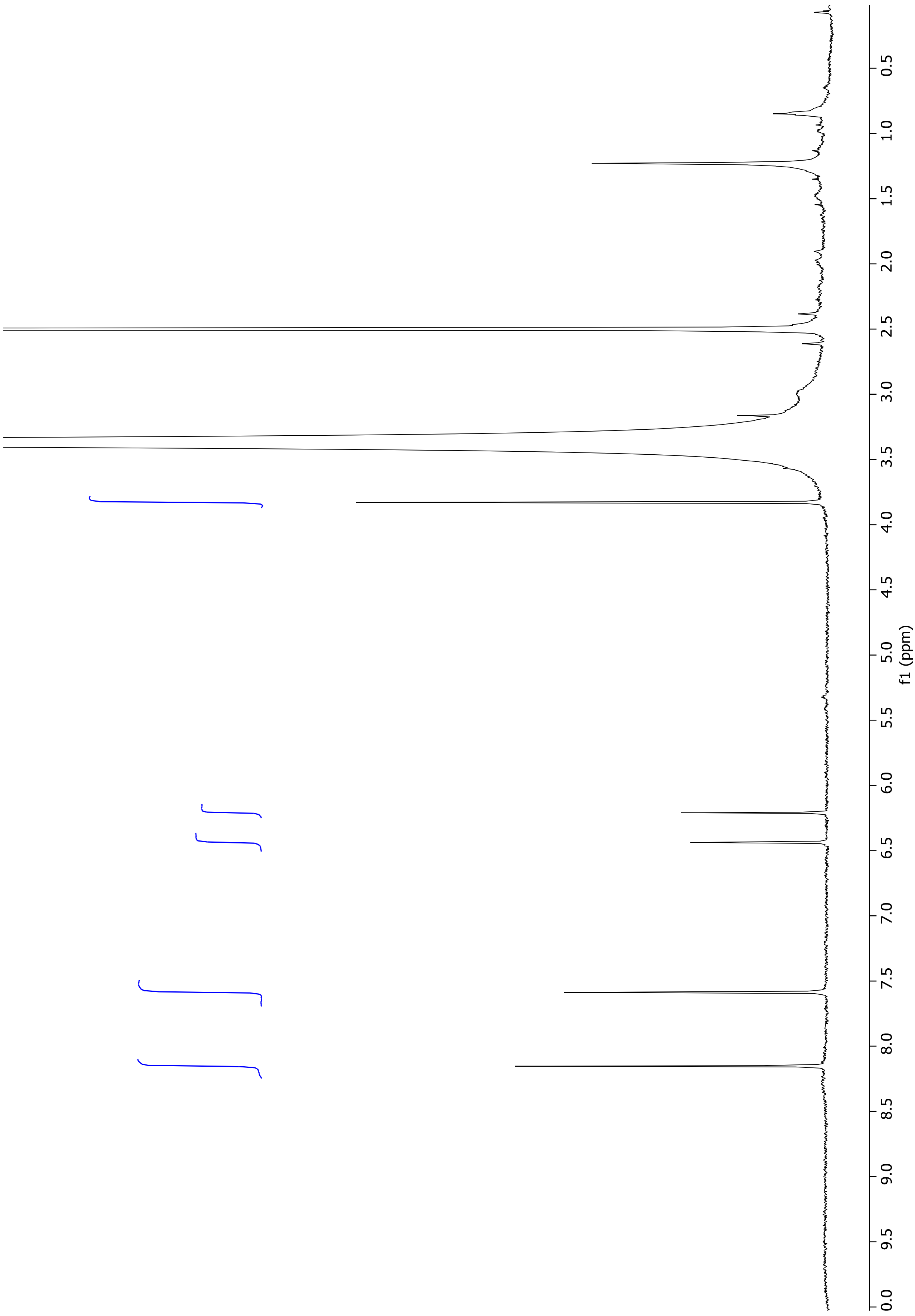

Figure S4 – ^{1}H NMR Spectrum (600 MHz, DMSO-d_6) of **3**

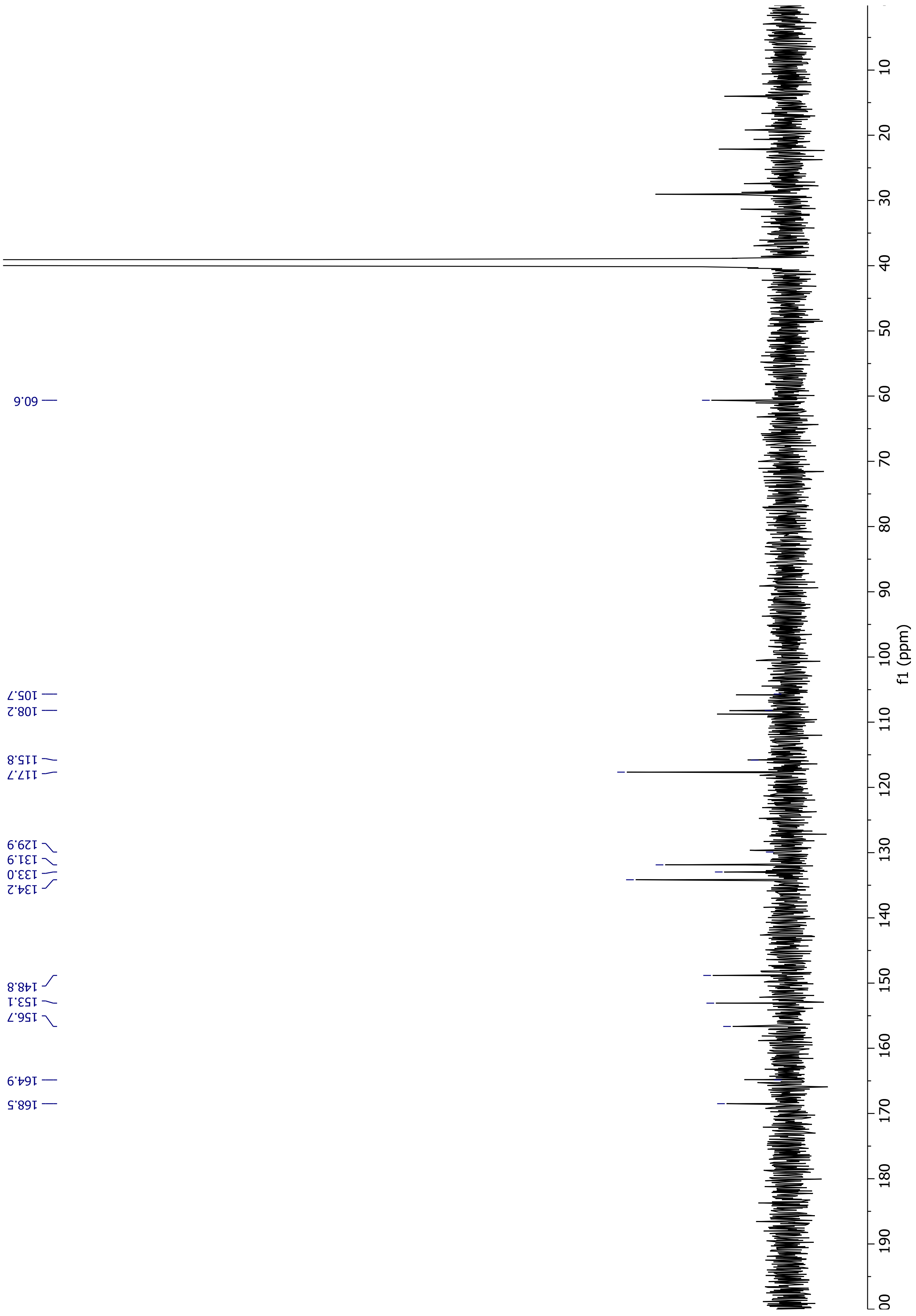

Figure S5 – ^{13}C NMR Spectrum (150 MHz, DMSO-d_6) of **3**

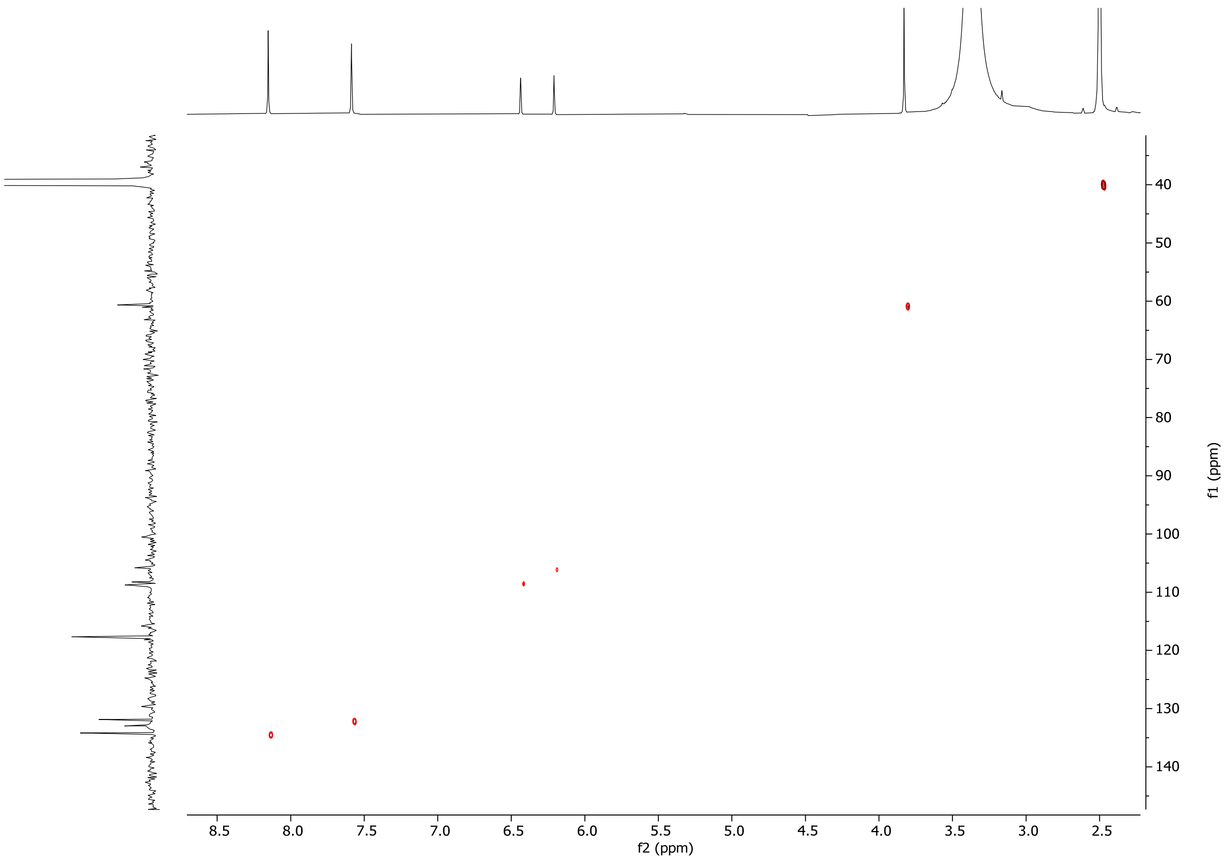

Figure S6 – HSQC NMR Spectrum (600 MHz, DMSO-d$_6$) of **3**

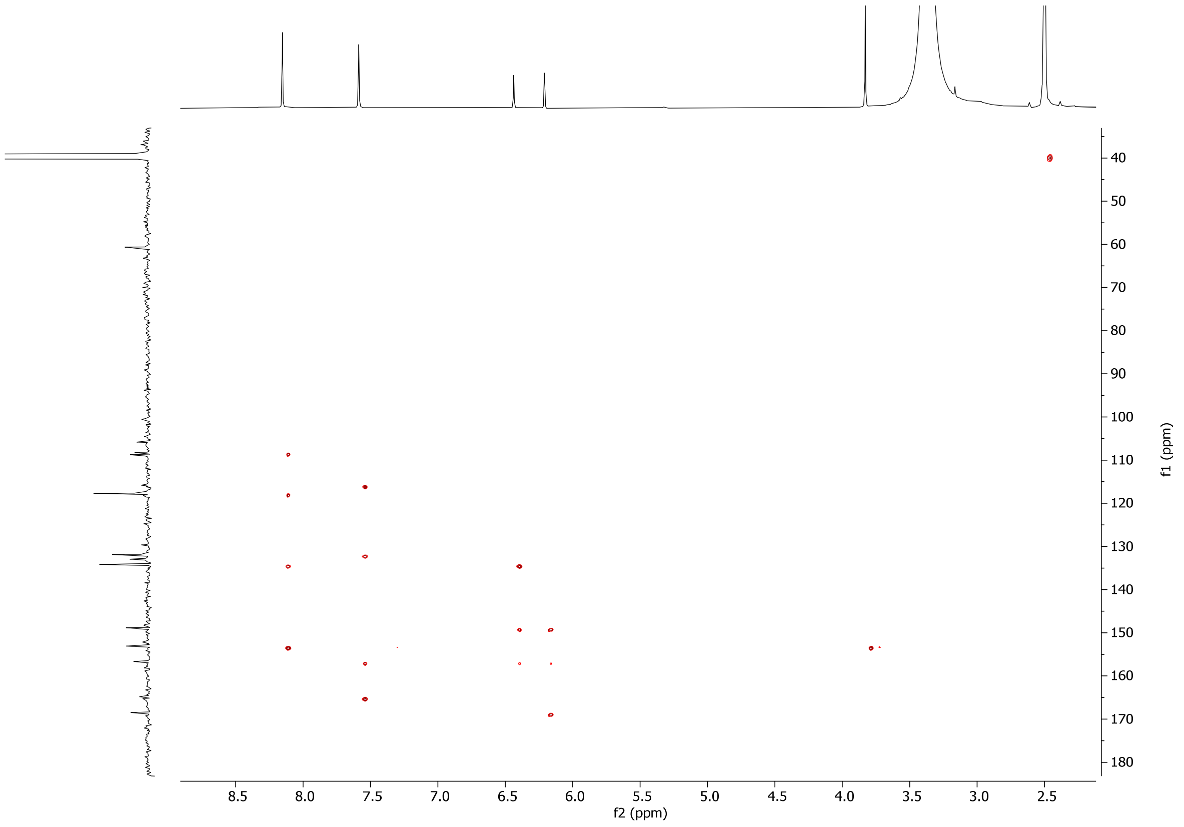

Figure S7 – HMBC NMR Spectrum (600 MHz, DMSO-d$_6$) of **3**

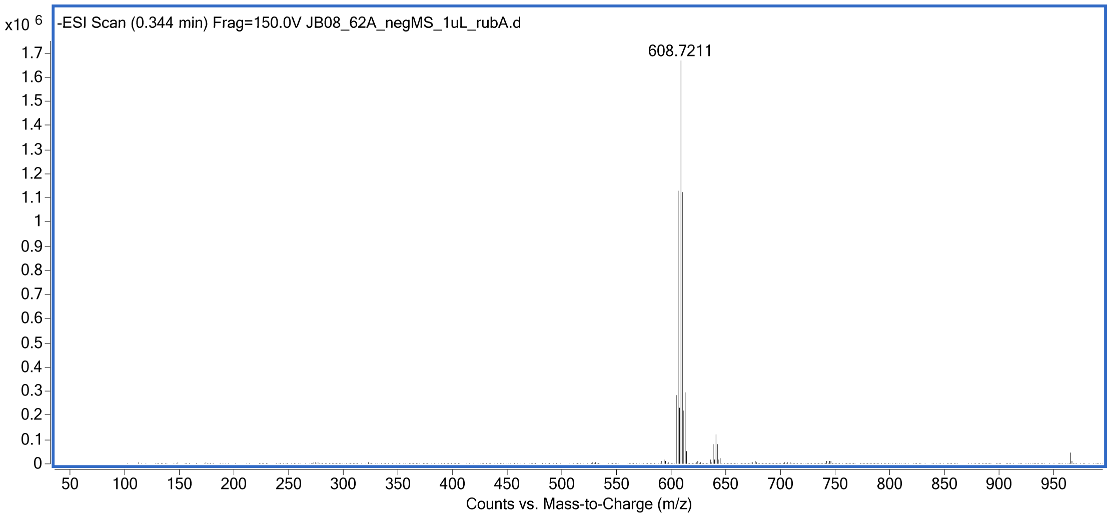

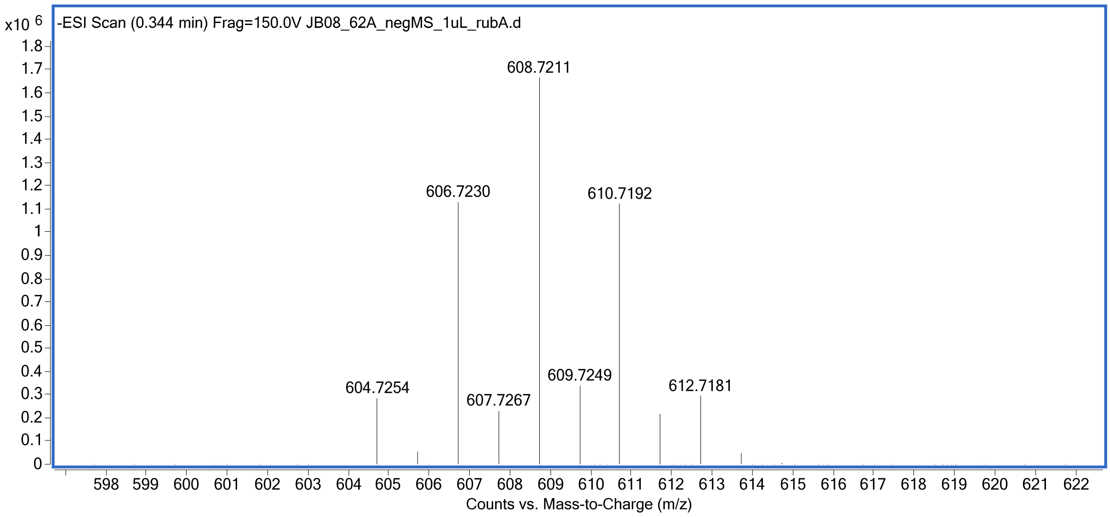

Figure S8 – (–)-HRESIMS spectra of **3**

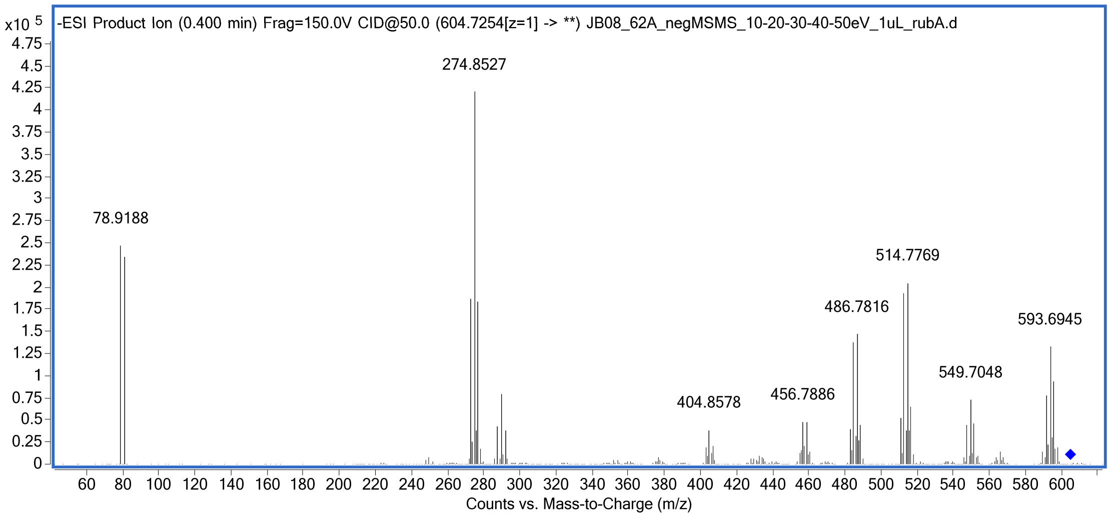

Figure S9 – (–)-HRESIMS/MS spectrum of **3**

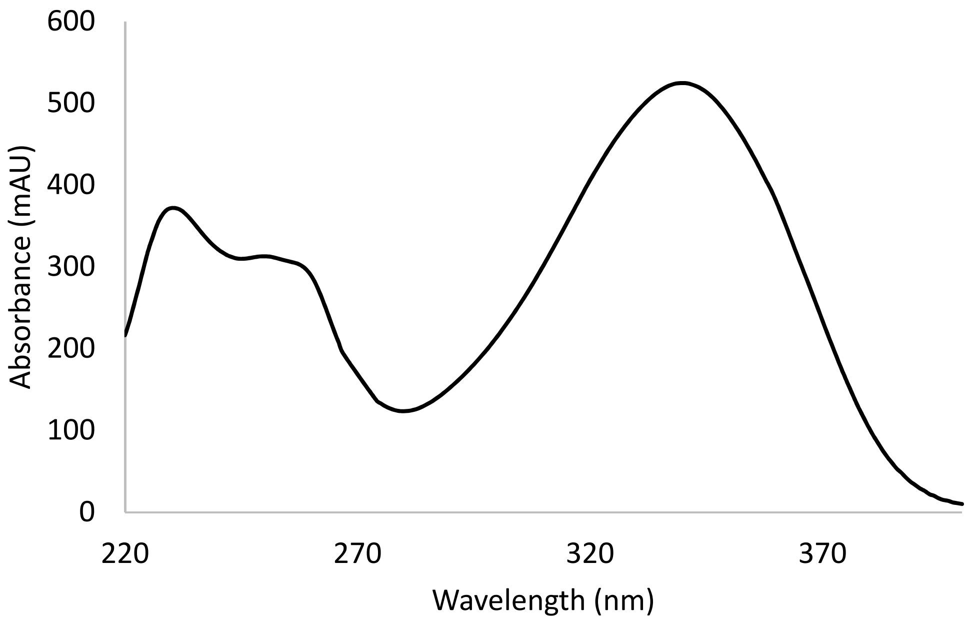

Figure S10 – UV/Vis spectrum of **3**

Table S2 – ^{13}C (150 MHz) and ^{1}H (600 MHz) NMR data for *Z*–Rubrolide U (*Z*–**4**) in DMSO–d_6.

position	^{13}C (δ)	^{1}H (δ, int., mult., *J* in Hz)	HMBC	ROESY
2	168.6			
3	105.0	6.13 (1H, s)	2, 5	2'/6'
4	156.8			
5	146.7			
6	110.0	6.37 (1H, s)	4, 5, 2'', 6''	2'/6', 2'', 6''
1'	131.6			
2'/6'	131.5	7.56 (2H, s)	4, 4', 3'/5'	3, 6
3'/5'	115.4			
4'	164.6			
1''	127.4			
2''	131.1	7.81 (1H, dd, 8.7, 2.2)	4'', 6''	6
3''	112.6	7.19 (1H, d, 8.7)	1'', 5''	CH_3O–4''
4''	155.3			
5''	110.7			
6''	134.1	8.11 (1H, d, 2.2)	4'', 5''	6
CH_3O–4''	56.4	3.90 (3H, s)	4''	3''

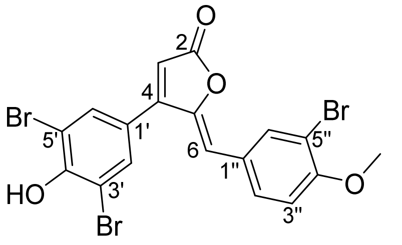

Z-**4**

Table S3 – ^{13}C (150 MHz) and ^{1}H (600 MHz) NMR data for *E*–Rubrolide U (*E*–**4**) in DMSO–d_6.

position	^{13}C (δ)	^{1}H (δ, int., mult., *J* in Hz)	HMBC	ROESY
2	168.1			
3	110.3	6.18 (1H, s)	2, 5	2’/6’
4	154.7			
5	147.6			
6	113.8	6.88 (1H, s)		2’’, 6’’
1’	not			
2’/6’	132.0	7.04 (2H, s)	4, 4’, 3’/5’	3, 2’’, 6’’
3’/5’	114.3			
4'	164.0			
1’’	126.5			
2’’	130.7	7.05 (1H, dd, 8.7, 2.2)		6, 2’/6’
3’’	111.9	6.87 (1H, d, 8.7)	1’’, 5’’	CH_3O–4’’
4'’	155.4			
5’’	110.3			
6’’	134.1	7.40 (1H, d, 2.2)		6, 2’/6’
CH_3O–4’’	56.2	3.79 (3H, s)	4’’	3’’

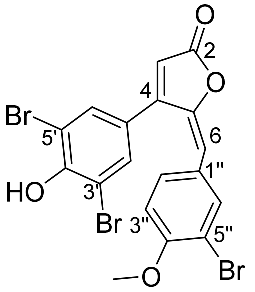

E-**4**

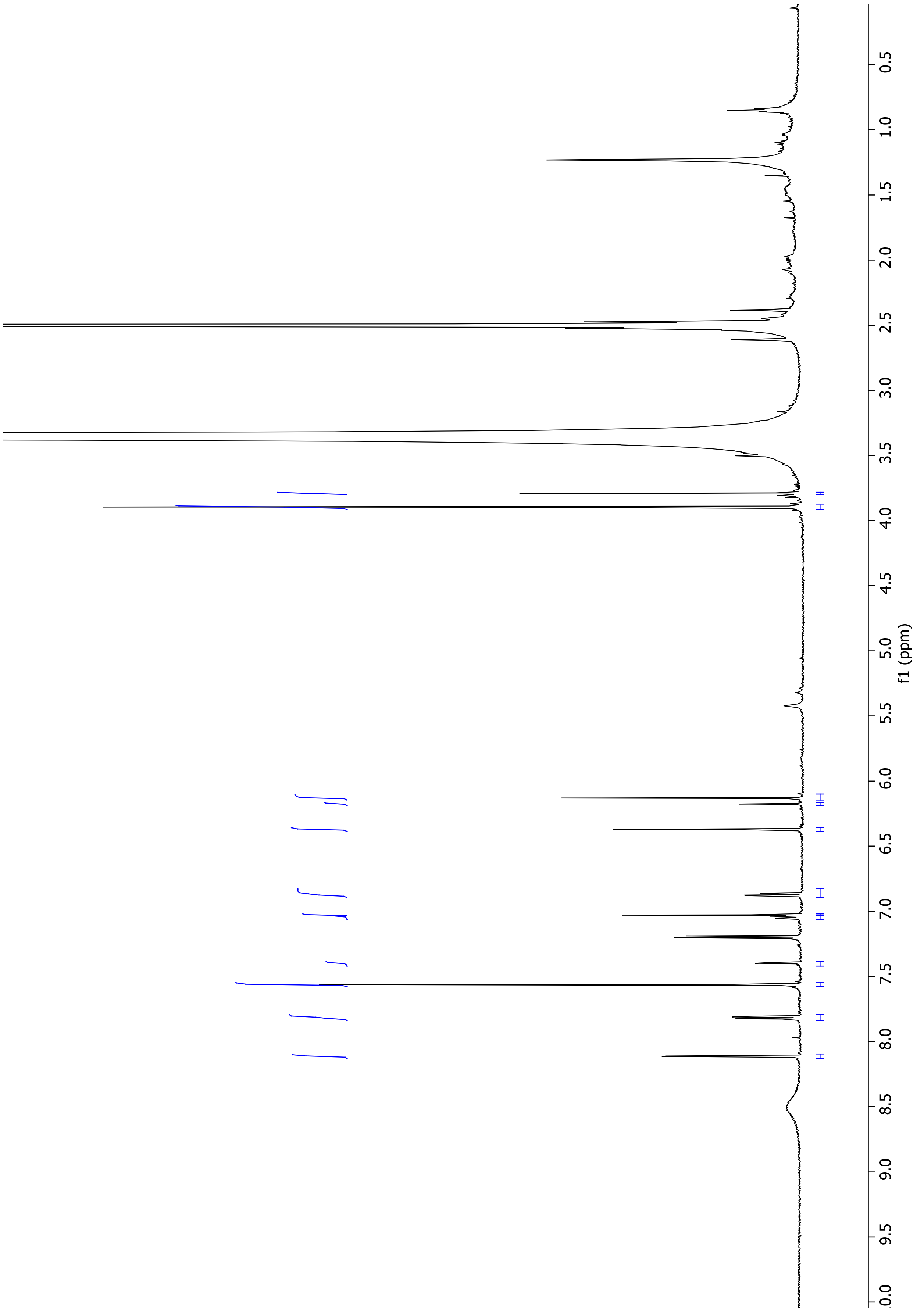

Figure S11 – ¹H NMR Spectrum (600 MHz, DMSO-d₆) of **4**

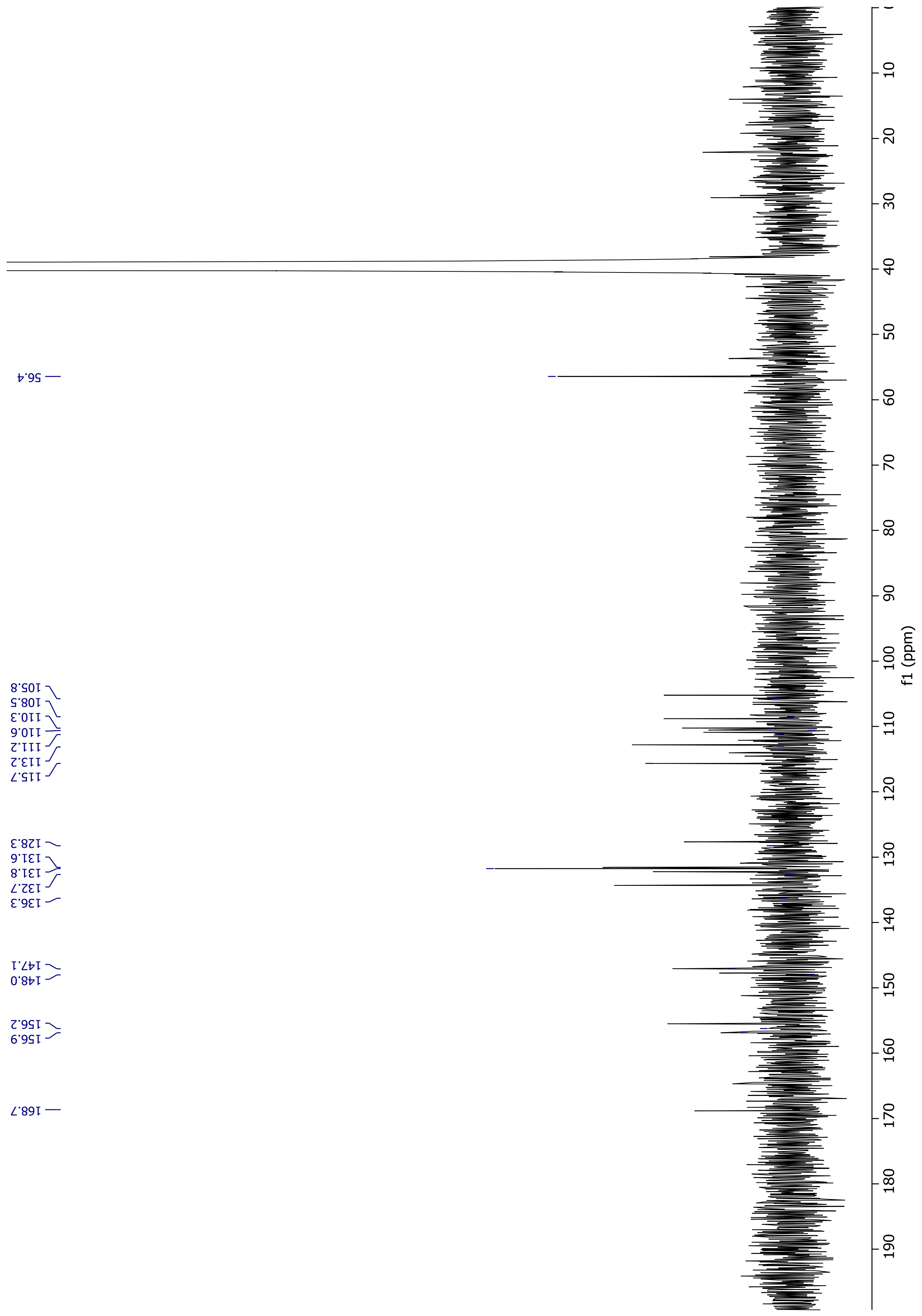

Figure S12 – ^{13}C NMR Spectrum (150 MHz, DMSO-d$_6$) of **4**

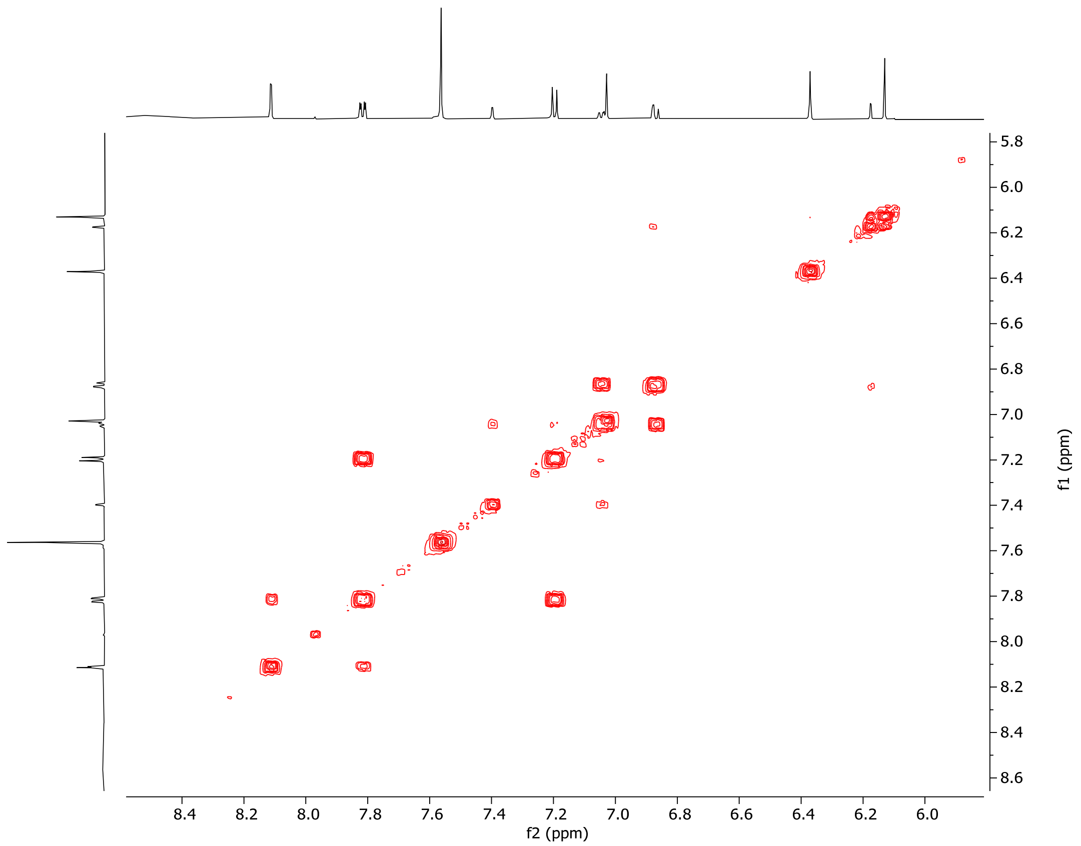

Figure S13 – COSY NMR Spectrum (600 MHz, DMSO-d6) of **4**

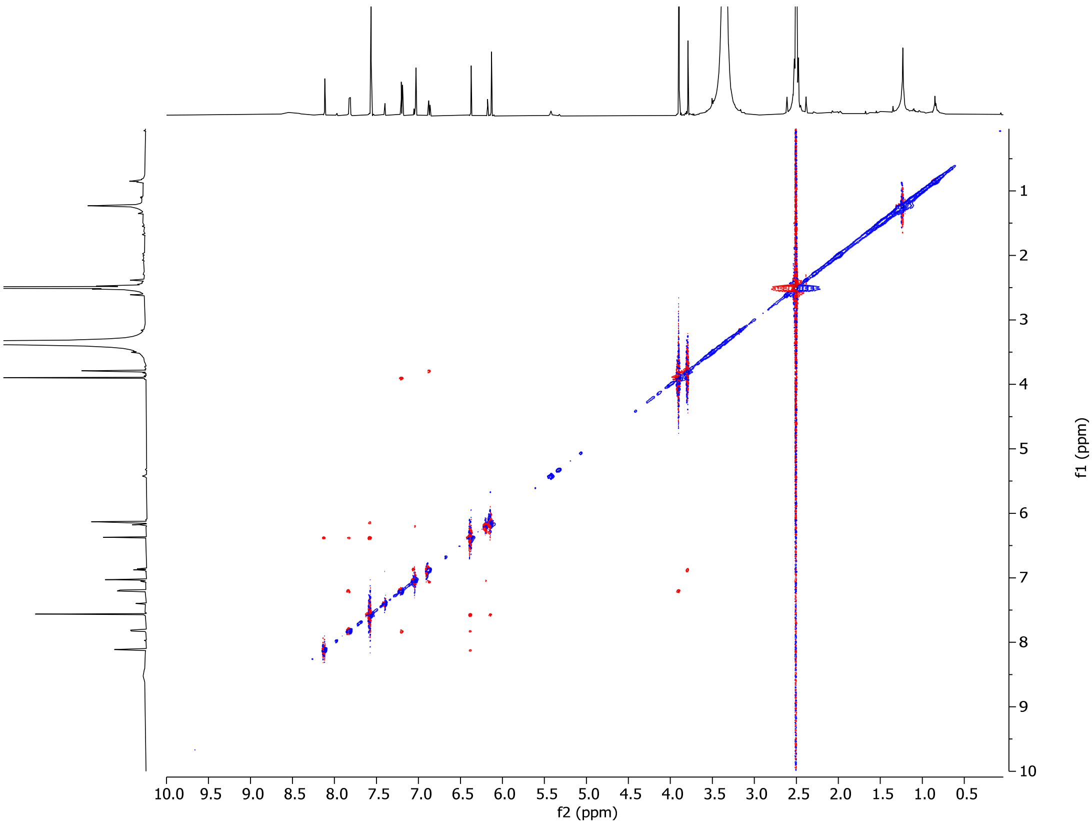

Figure S14 – ROESY NMR Spectrum (600 MHz, DMSO-d6) of **4**

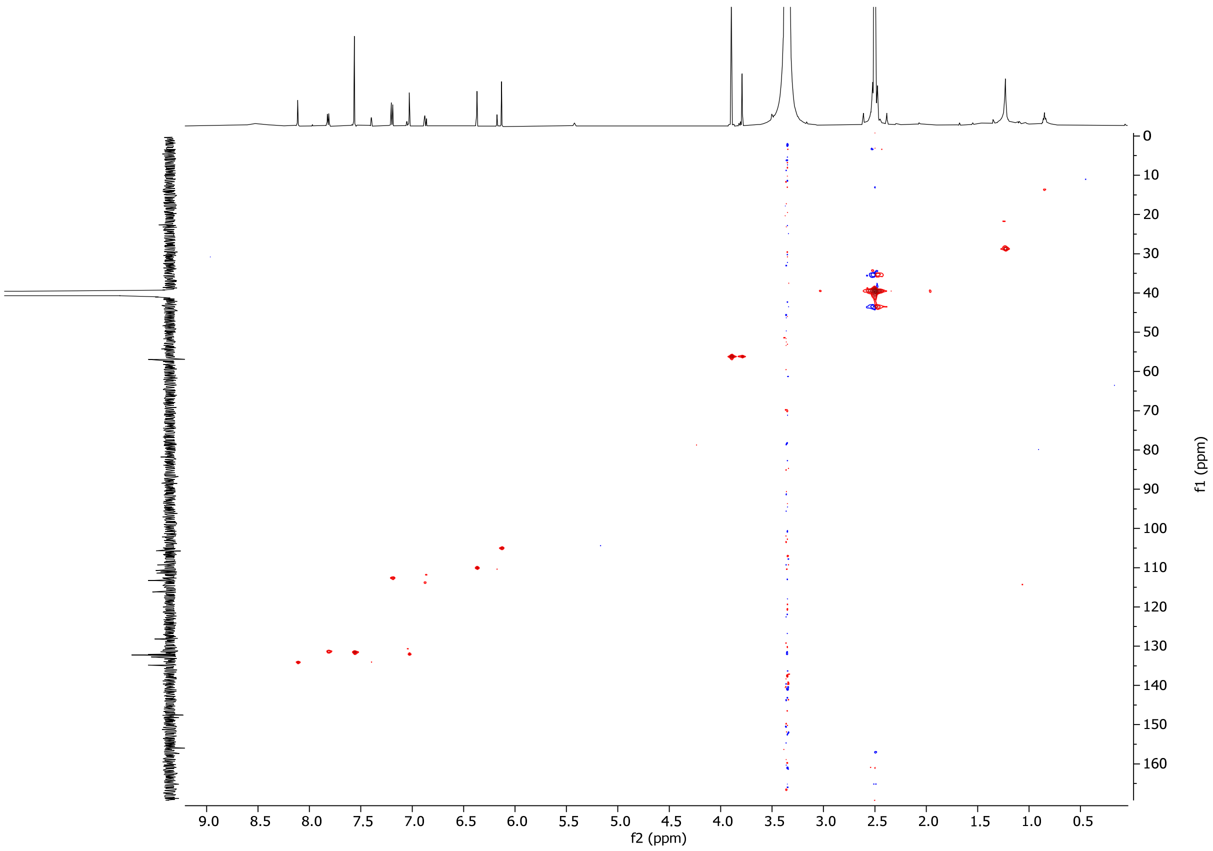

Figure S15 – HSQC NMR Spectrum (600 MHz, DMSO-d_6) of **4**

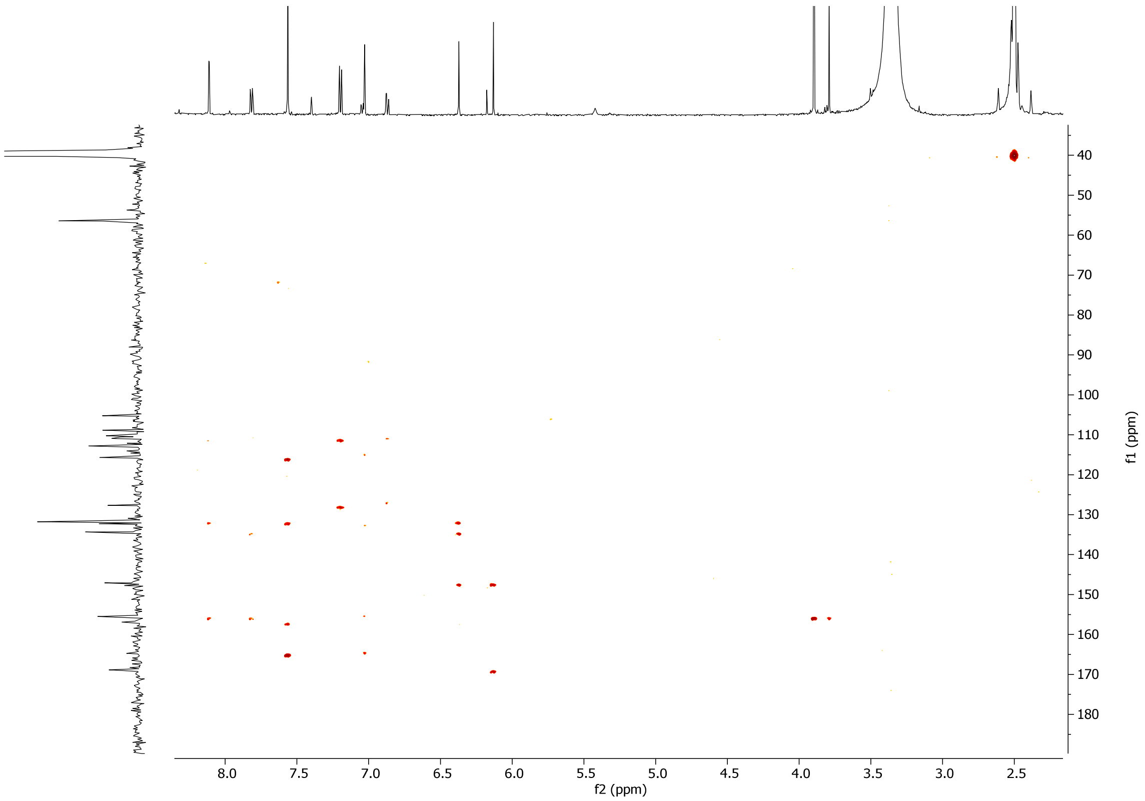

Figure S16 – HMBC NMR Spectrum (600 MHz, DMSO-d_6) of **4**

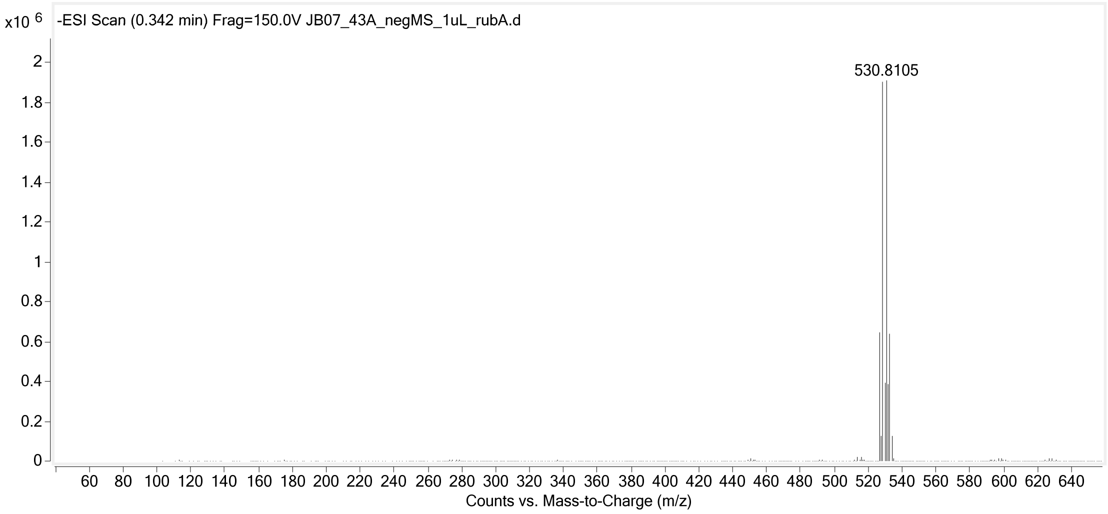

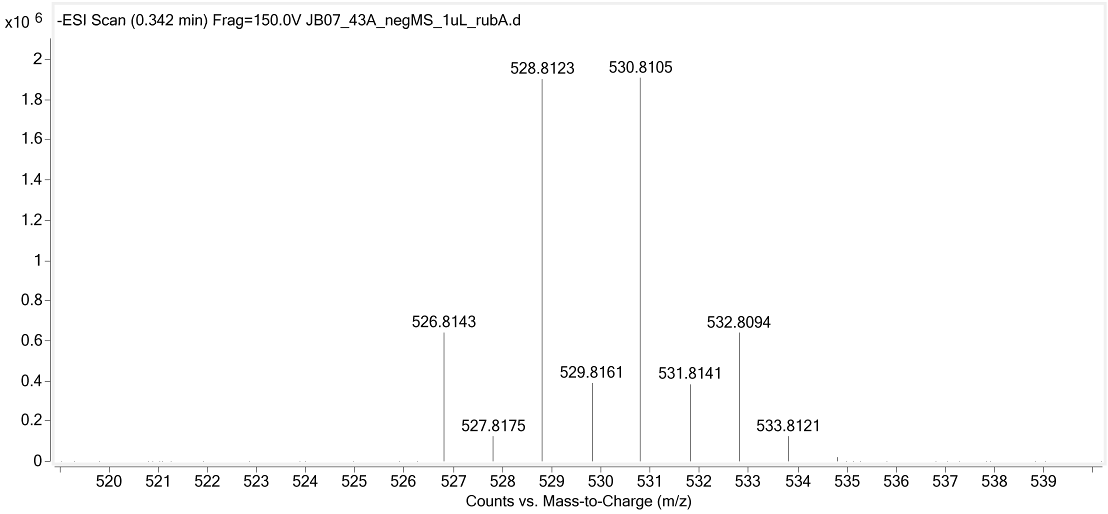

Figure S17 – (–)-HRESIMS spectra of **4**

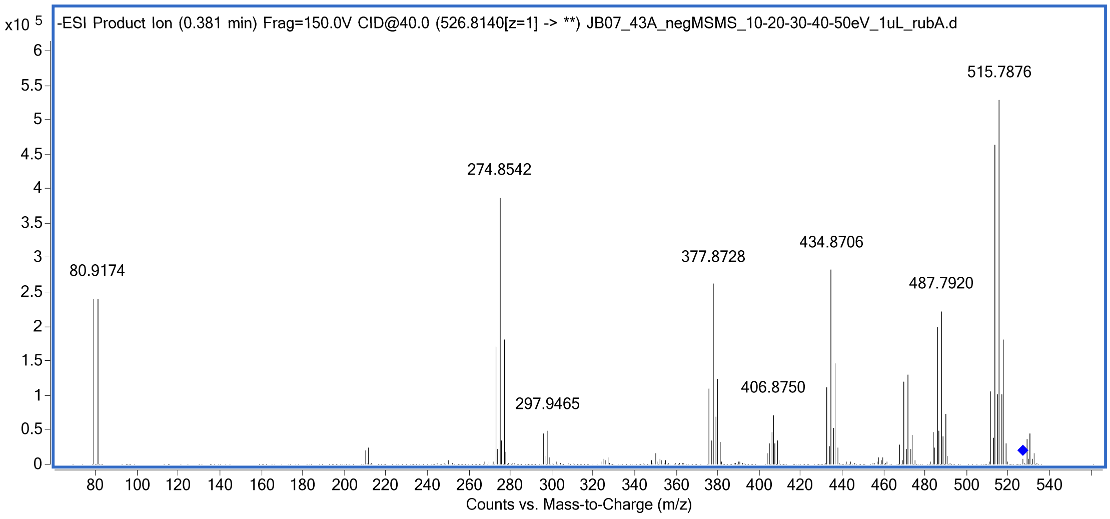

Figure S18 – (–)-HRESIMS/MS spectrum of **4**

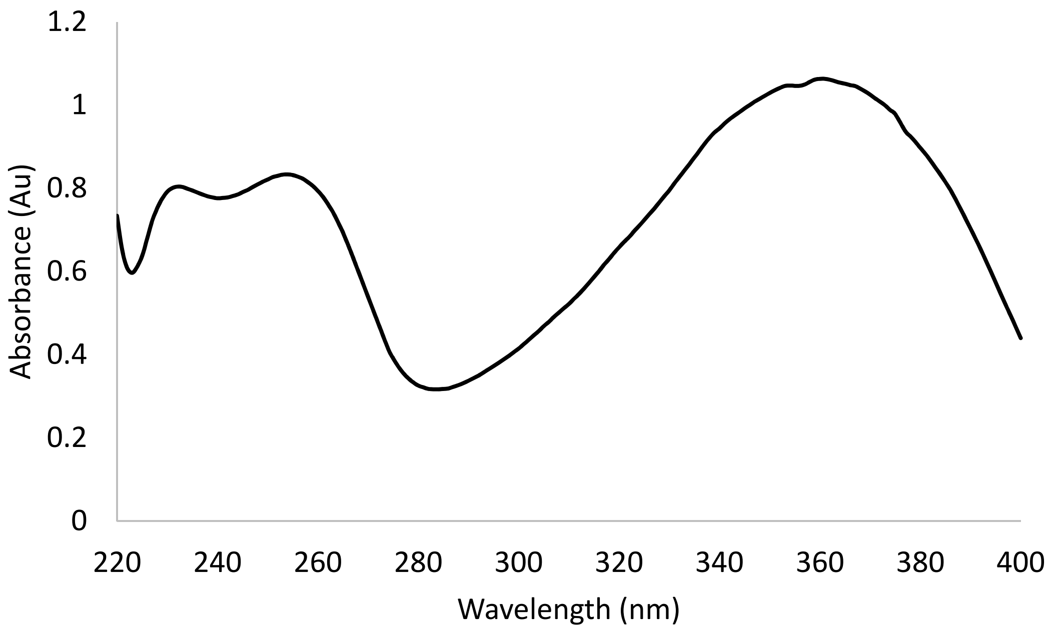

Figure S19 – UV/Vis spectrum of **4**
